# Predictive models of objective oropharyngeal OSA surgery outcomes: Success rate and AHI reduction ratio

**DOI:** 10.1371/journal.pone.0185201

**Published:** 2017-09-22

**Authors:** Ji Ho Choi, Jae Yong Lee, Jaehyung Cha, Kangwoo Kim, Seung-No Hong, Seung Hoon Lee

**Affiliations:** 1 Department of Otorhinolaryngology-Head and Neck Surgery, Soonchunhyang University College of Medicine, Bucheon Hospital, Bucheon, Korea; 2 Medical Science Research Center, Korea University College of Medicine, Seoul, Korea; 3 Department of Otorhinolaryngology-Head and Neck Surgery, Korea University College of Medicine, Ansan Hospital, Ansan, Korea; Technische Universitat Munchen, GERMANY

## Abstract

**Objective:**

The aim of this study was to develop a predictive model of objective oropharyngeal obstructive sleep apnea (OSA) surgery outcomes including success rate and apnea-hypopnea index (AHI) reduction ratio in adult OSA patients.

**Study design:**

Retrospective outcome research.

**Methods:**

All subjects with OSA who underwent oropharyngeal and/or nasal surgery and were followed for at least 3 months were enrolled in this study. Demographic, anatomical [tonsil size (TS) and palate-tongue position (PTP) grade (Gr)], and polysomnographic parameters were analyzed. The AHI reduction ratio (%) was defined as [(postoperative AHI—preoperative AHI) x 100 / postoperative AHI], and surgical success was defined as a ≥ 50% reduction in preoperative AHI with a postoperative AHI < 20.

**Results:**

A total of 156 consecutive OSAS adult patients (mean age ± SD = 38.9 ± 9.6, M / F = 149 / 7) were included in this study. The best predictive equation by Forward Selection likelihood ratio (LR) logistic regression analysis was:

$$\text{ln}\left(\frac{P_{x}}{1-P_{x}}\right)=1.518-0.039\times \text{Age}+1.392\times \text{TS}\;\text{Gr}-0.803\times \text{PTP}\;\text{Gr}$$

The best predictive equation according to stepwise multiple linear regression analysis was:

$$\begin{array}{l} \text{AHI}\;\text{reduction}\;\text{ratio}=-39.464+(32.752\times \text{TS}\;\text{Gr})+(2.623\times \text{AHI})-(2.542\times \text{Arousal}\;\text{index})\\ +[1.245\times \text{Minimum}\;\text{Sa}{\text{O}}_{2}(\%)]-[0.599\times \text{Snoring}\;(\%)] \end{array}$$

(TS/PTP Gr = 1 if TS/PTP Gr 3 or 4, TS/PTP Gr = 0 if TS/PTP Gr 1 or 2)

**Conclusion:**

The predictive models for oropharyngeal surgery described in this study may be useful for planning surgical treatments and improving objective outcomes in adult OSA patients.

## Introduction

Obstructive sleep apnea (OSA) is characterized by repeated episodes of a significant reduction or complete cessation in breathing during sleep, and is caused by a narrowing or obstruction of the upper airway, including the nasal cavity, pharynx and larynx.[1] If detection or management is neglected, OSA can lead to various symptoms including excessive daytime sleepiness and serious consequences such as cardiovascular disease.[2, 3] Therefore, prompt diagnosis and optimal treatment for OSA is important for improving patient health. There are a variety of therapeutic options for OSA such as positional therapy, surgery, weight loss, positive airway pressure (PAP), and oral appliance (OA). [4] In general, the optimal treatment method is determined according to the patient’s anatomical structures (e.g., tongue, tonsil, soft palate, adenoid, nasal septum, inferior turbinate), polysomnographic results (e.g., apnea-hypopnea index [AHI], arterial oxygen saturation [SaO_2_]) and personal preferences.[5]

Surgical procedures for the management of sleep-disordered breathing (SDB) have been performed since the 1960’s.[6] Based on anticipated sites of obstruction, a diverse array of surgical modifications of the upper airway can be carried for patients, including nasal surgery (e.g., septoplasty, turbinate surgery) oropharyngeal procedures (e.g., tonsillectomy, uvulopalatopharyngoplasty [UPPP], uvulopalatal flap [UPF]) and hypopharyngeal procedures (e.g., genioglossus advancement, radiofrequency tongue base ablation, partial glossectomy).[7, 8] Of these, oropharyngeal surgery such as UPPP is one of the most frequently used operative techniques for OSA and is sometimes performed with nasal surgery and/or hypopharyngeal procedure depending on the patient’s level of obstruction.[7, 8]

Surgery carries with it various advantages and disadvantages. One of the main drawbacks of upper airway surgery is the difficulty in predicting the treatment outcome.[5, 7, 8] According to a review by Sher et al., the success rate of UPPP is as low as 40.7% when performed randomly in adult patients with OSA.[9] Since the early 2000’s, several studies have been carried out to improve objective outcomes of surgical management based on anatomy.[10–12] As a result, anatomical structures such as tonsil size and palate-tongue position appear to be helpful for predicting the success rate of UPPP, suggesting that anatomy is more useful than severity of OSA in predicting surgical outcomes.[10–12]

A recent systematic review and meta-analysis by the American Academy of Sleep Medicine (AASM) evaluated objective surgical outcomes using the AHI reduction ratio, which refers to the extent to which the mean postoperative AHI decreases compared to the mean preoperative AHI.[8] In that study, the mean AHI reduction ratio was 33% (95% confidence interval [CI] 23% to 42%) after UPPP.[8] However, there is still insufficient data to investigate the effect of oropharyngeal OSA surgery on AHI reduction according to anatomical factors, as well as to assess the difference between success rate and AHI reduction ratio following surgical therapy. In addition, there is little literature on the development of predictive models based on objectively analyzing oropharyngeal OSA surgery outcomes. Therefore, the purpose of the present study was to 1) estimate the effect of oropharyngeal OSA surgery on objective outcomes such as success rates and AHI reduction ratio, 2) compare success rates and AHI reduction ratios according to anatomical structures including tonsil size and palate-tongue position, and 3) develop predictive equation-based models for determining outcomes including success rates and AHI reduction ratios before oropharyngeal OSA surgery in adult patients with OSA.

## Materials and methods

### Subjects

This retrospective study was reviewed and approved by the Institutional Review Board of Korea University Ansan Hospital, and informed consent was waived. Inclusion criteria were as follows: subjects who 1) were 18 years of age or older; 2) had diverse OSA symptoms including excessive daytime sleepiness, habitual snoring, and observed sleep apnea; 3) were diagnosed with OSA (AHI ≥ 5) based on the International Classification of Sleep Disorders (ICSD-2, 2nd ed.) after standard polysomnography[13]; 4) failed or refused to use a medical device such as a PAP or OA; 5) underwent oropharyngeal OSA surgery (e.g., UPPP, UPF, tonsillectomy) with/without nasal surgery (e.g., septoplasty, turbinate surgery, endoscopic sinus surgery); and 6) completed postoperative standard polysomnography at a 3 month follow-up. Exclusion criteria were as follows: subjects who had 1) a medical history of critical cardiopulmonary disease (e.g., congestive heart failure, chronic obstructive pulmonary disease) or sleep disorder (e.g., central sleep apnea syndrome, hypoventilation syndrome); 2) a history of previous oropharyngeal OSA surgery; 3) morbid obesity (a body mass index [BMI] greater than 40 kg/m^2^); and 4) other significant conditions (genetic syndrome, neuromuscular disease, craniofacial abnormality).

### Physical examinations

All subjects underwent an upper airway inspection and were evaluated using the anatomy-based staging system previously developed by Friedman et al.[10, 11] Tonsil size (TS) and palate-tongue position (PTP) were classified from 1 to 4. All patients were staged according to an anatomy-based (Friedman) staging system and modified anatomy-based staging system (Table 1). The two staging systems were different in that stage II in the original staging system is divided into two sub-stages in the modified staging system, namely IIa and IIb. Subjects with morbid obesity (BMI > 40 kg/m^2^) were not considered in this study based on the exclusion criteria.

**Table 1:** Definition of anatomical factors and staging systems.

	Definition
**Tonsil size (TS)**	
Grade (Gr) 1	Tonsils hidden within the pillars
Gr 2	Tonsils extending to the pillars
Gr 3	Tonsils beyond the pillars but not to the midline
Gr 4	Tonsils that extend to the midline
**Palate-Tongue Position (PTP)**	
Gr 1	Entire uvula and tonsils or pillars are clearly visible
Gr 2	The uvula but not tonsils are visible
Gr 3	Only the soft palate is visible
Gr 4	Only the hard palate is visible
**Anatomy-based (Friedman) staging system**	
Stage I	TS G 3 or 4 and PTP G 1 or 2
Stage II	TS G 3 or 4 and PTP G 3 or 4
	TS G 1 or 2 and PTP G 1 or 2
Stage III	TS G 1 or 2 and PTP G 3 or 4
**Modified anatomy-based staging system**	
Stage I	TS G 3 or 4 and PTP G 1 or 2
Stage IIa	TS G 3 or 4 and PTP G 3 or 4
Stage IIb	TS G 1 or 2 and PTP G 1 or 2
Stage III	TS G 1 or 2 and PTP G 3 or 4

### Sleep study

An attended nocturnal standard polysomnography was performed in all subjects using a computerized polysomnographic system (Alice 4; Respironics, Atlanta, GA, USA). The measured general parameters included electroencephalogram, electrooculogram, chin and leg electromyogram, airflow and respiratory effort signals, oxygen saturation, body position, and electrocardiogram. A sleep technician conducted nocturnal monitoring of all subjects and manual scoring of all sleep data based on the AASM scoring manual.[14]

### Surgery and surgical outcomes

All subjects were treated with oropharyngeal OSA surgery and/or nasal surgery. In this study, oropharyngeal OSA surgery included modified UPPP (uvula preserving technique), UPF, and Tonsillectomy.[15, 16] Nasal surgery included septoplasty, turbinate surgery, and endoscopic sinus surgery. All surgical modifications of the upper airway were performed under general anesthesia.

Objective surgical outcomes were assessed by two methods, namely, surgical success rate and AHI reduction ratio. Surgical success was defined as a postoperative AHI < 20 and a ≥ 50% reduction in preoperative AHI.[17] The AHI reduction ratio (%) was defined as [(postoperative AHI—preoperative AHI) x 100 / postoperative AHI].[8]

### Statistics

Data are expressed as frequencies (percent) for categorical variables, and as the means ± standard deviation (SD) for continuous variables. In comparison between oropharyngeal OSA surgery alone and oropharyngeal OSA with nasal surgery, *P*-values were calculated by Student's t-test or Mann-Whitney U test for continuous variables and chi-square test for categorical variables. The Chi-square test was used to compare surgical success rates among stages. ANOVA followed by a post hoc test (Dunnett's test) was used to compare mean AHI reduction ratios among stages. Forward Selection [Likelihood Ratio (LR)] in logistic regression analysis was used to develop the best predictive equation model for predicting success. Stepwise multiple linear regression analysis was used to obtain the best predictive equation model for the AHI reduction ratio. Pearson’s correlation coefficient was used to examine the associations between AHI reduction ratio and predicted AHI reduction ratio. SPSS version 20.0 statistical software (SPSS Inc., Chicago, IL, USA) was used for statistical analysis of all the data. *P*-values < 0.05 were deemed statistically significant.

## Results

### Subjects

A total of 156 consecutive OSAS adult patients (M / F = 149 / 7) were included in the final study. The mean patient age was 38.9 ± 9.6 years old and mean BMI was 27.5 ± 3.1 kg/m^2^. Baseline data including tonsil size, palate-tongue position, and polysomnographic parameters were summarized in Table 2. UPPP (n = 137, 87.8%) was performed in most patients, followed by UPF (n = 16, 10.3%) and tonsillectomy (n = 3, 1.9%).

**Table 2:** Baseline data (N = 156).

	Subjects (N = 156)
**Demographic parameters**	
Age (years)	38.9 ± 9.6
Sex (male/female)	149 / 7
Body mass index (kg/m^2^)	27.5 ± 3.1
**Anatomical parameters**	
Tonsil size grade	2.3 ± 0.9
Palate-tongue position grade	2.3 ± 0.7
**Polysomnographic parameters**	
Apnea-hypopnea index (events/hour of TST)	38.3 ± 25.1
Arousal index (events/hour of TST)	44.7 ± 20.4
Minimum SaO_2_ (%)	76.5 ± 11.3
Snoring (% of TST)	27.5 ± 17.7

Data are means ± SD.

TST, total sleep time; SaO_2_, arterial oxygen saturation.

### Objective surgical outcomes

#### Effect of nasal surgery on surgical outcomes

Comparative outcomes between oropharyngeal OSA surgery alone (n = 50) and oropharyngeal OSA surgery (n = 106) with nasal surgery were presented in Table 3. There was no statistical difference in demographic (age, sex, and BMI), anatomical (tonsil size grade and palate-tongue position grade) and polysomnographic (preoperative AHI, postoperative AHI, and AHI reduction ratio) parameters.

**Table 3:** Comparison between oropharyngeal OSA surgery alone and oropharyngeal OSA with nasal surgery groups.

Variable	Oropharyngeal OSA surgery alone group (n = 50)	Oropharyngeal OSA surgery with nasal surgery group (n = 106)	*P*-value
**Demographic parameters**			
Age (years)	40.0 ± 11.4	38.4 ± 8.5	0.356
Sex (male/female)	45 / 5	104 / 2	0.061
Body mass index (㎏/㎡)	27.7 ± 3.3	27.4 ± 3.1	0.497
**Anatomical parameters**			
Tonsil size grade	2.3 ± 1.0	2.3 ± 0.8	0.732
Palate-tongue position grade	2.3 ± 0.8	2.3 ± 0.7	0.636
**Polysomnographic parameters**			
Preoperative AHI (events/hour of TST)	35.6 ± 23.1	39.6 ± 25.9	0.439
Postoperative AHI (events/hour of TST)	17.1 ± 20.2	18.2 ± 19.5	0.668
AHI reduction ratio	36.3 ± 75.0	42.8 ± 62.8	0.897

Data are means ± SD.

TST, total sleep time; AHI, apnea-hypopnea index.

#### Success rate

**Fig 1:**
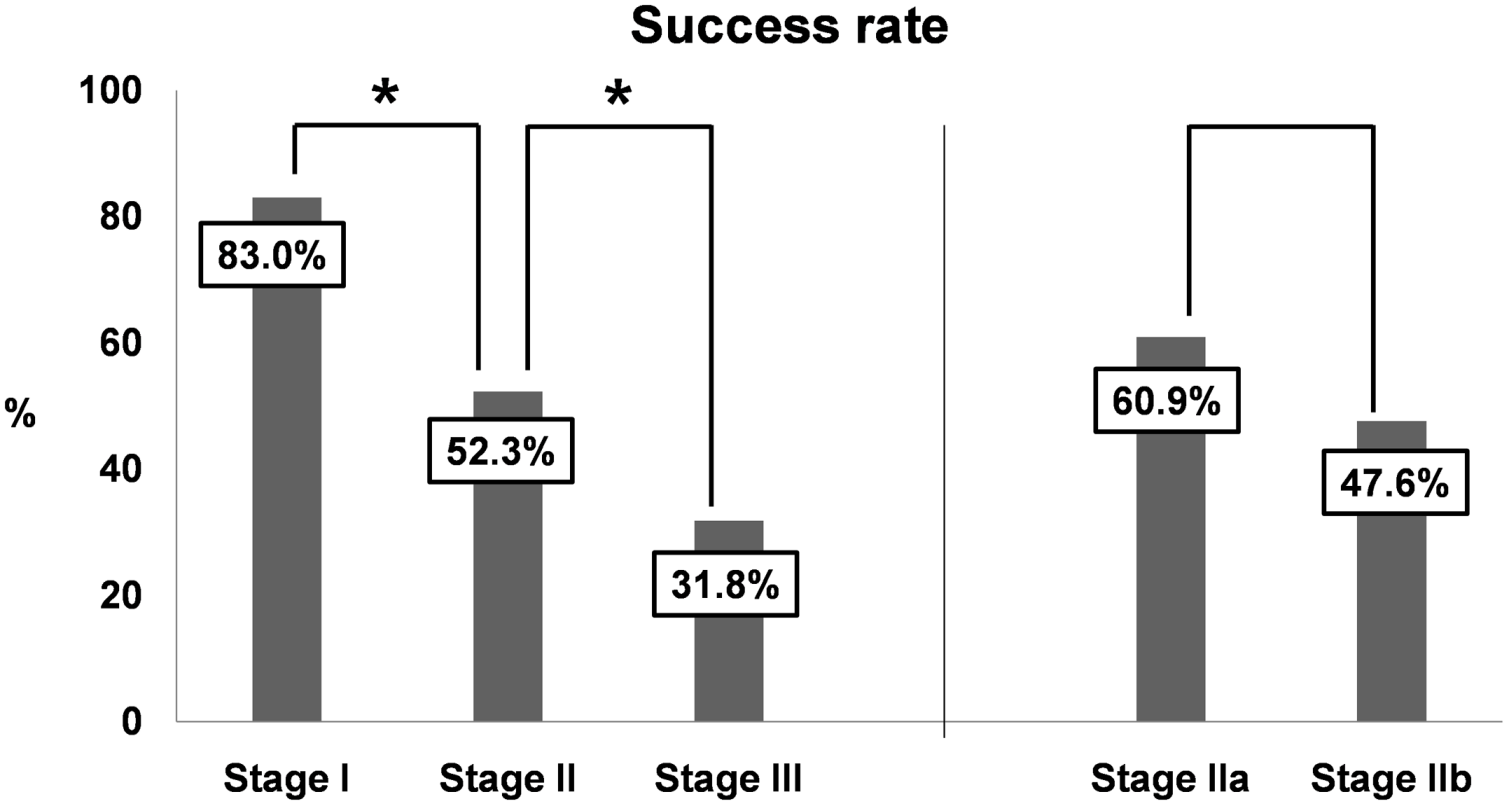
Success rates after oropharyngeal OSA surgery according to stage (N = 156). A significant difference was observed in success rate between stages I and II (P = 0.001). There was a significant difference in success rate between II and III (P = 0.034). However, there was no difference in the success rate between stages IIa and IIb (P = 0.306). * *P*< 0.05.

The overall success rate was 55.8% (87/156) and the surgical success rates in stages I, II (IIa and IIb), and III were 83.0% (39/47), 52.3% (34/65) [60.9% (14/23) and 47.6% (20/42)], and 31.8% (14/44), respectively (Fig 1). There were significant differences in the success rates with respect to stages I, II, and III, respectively [I and II (P = 0.001), II and III (P = 0.034), and I and III (P < 0.001)] However, no difference was noted in the success rate between stage IIa and IIb (P = 0.306).

#### AHI reduction ratio

**Fig 2:**
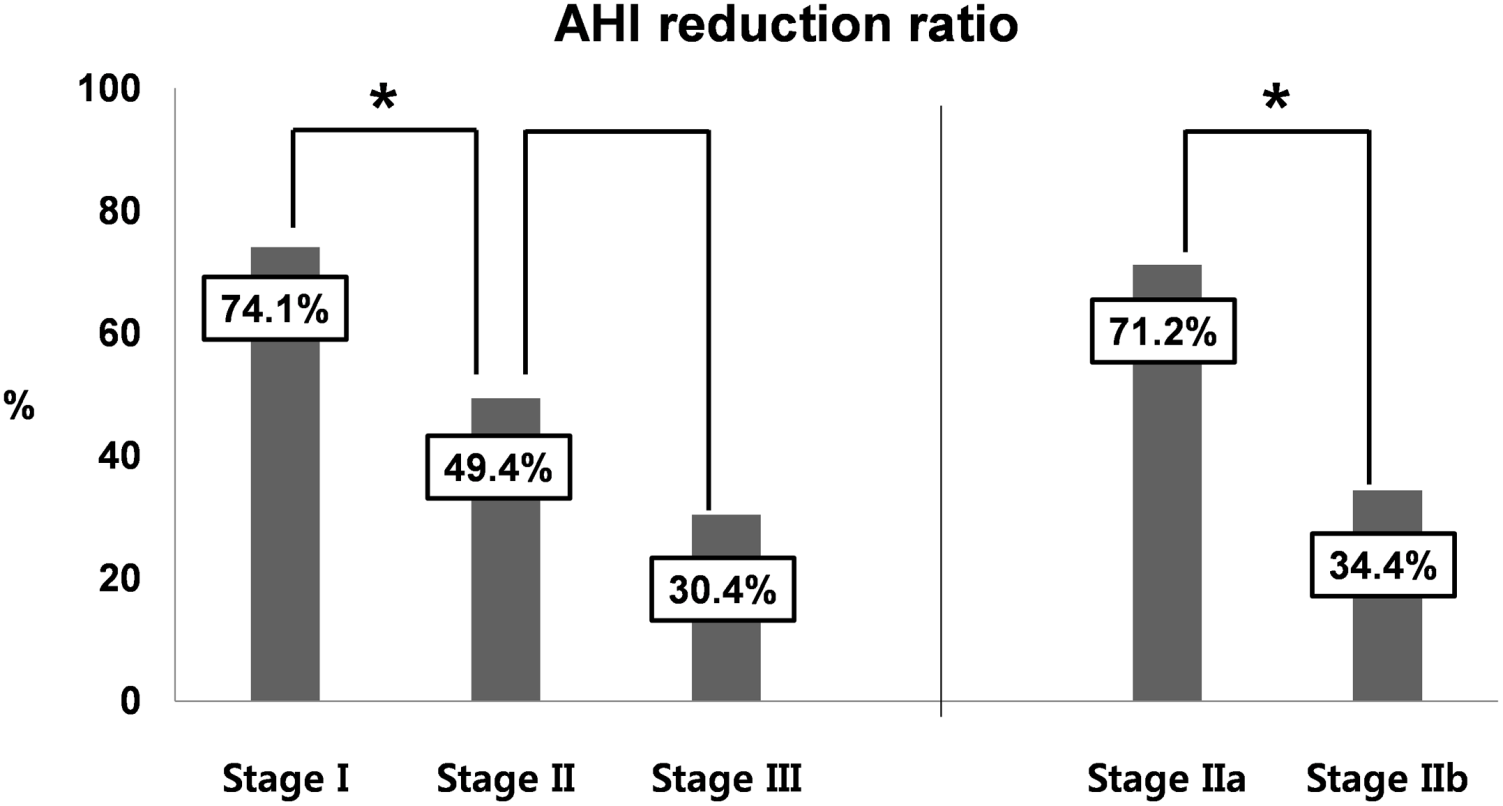
AHI reduction ratios after oropharyngeal OSA surgery according to stage (N = 156). A significant difference was noted in the AHI reduction ratio between stages I and II (P < 0.001), whereas no significant difference was found in the AHI reduction ratio between stages II and III (P = 0.827). A significant difference was identified for the AHI reduction ratio between stages IIa and IIb (P = 0.032). * *P*< 0.05.

The overall AHI reduction ratio was 53.5% (20.5/38.3), and the AHI reduction ratios for stages I, II (IIa and IIb), and III were 74.1% (35.1/47.4), 49.4% (16.0/32.4) [71.2% (27.0/37.9) and 34.4% (10.1/29.4)], and 30.4% (11.3/37.2), respectively (Fig 2). There was a significant difference in AHI reduction ratio between stages I and II (P < 0.001), whereas there was no significant difference in AHI reduction ratio between stages II and III (P = 0.827). A significant difference between stages I and III was noted (P < 0.001), and the difference in AHI reduction ratio between stages IIa and IIb was significant as well (P = 0.032).

### Prediction of objective surgical outcomes

#### Success rate

Baseline variables including age, BMI, TS Gr, PTP Gr, AHI, ArI, Min SaO_2_, and snoring were considered for the fitted equation. The best predictive equation by Forward Selection (LR) in logistic regression analysis was:

$$\text{ln}\left(\frac{P_{x}}{1-P_{x}}\right)=1.518-0.039\times \text{Age}+1.392\times \text{TS}\;\text{Gr}-0.803\times \text{PTP}\;\text{Gr}$$

TS Gr = 1 if TS Gr 3 or 4, TS Gr = 0 if TS Gr 1 or 2

PTP Gr = 1 if PTP Gr 3 or 4, PTP Gr = 0 if PTP Gr 1 or 2

The predicted success rates based on this equation model are shown in Table 4.

**Table 4:** Predicted success rates based on the best predictive equation model.

	20 years old	30 years old	40 years old	50 years old	60 years old
**Stage I**	89.4%	85.1%	79.4%	72.3%	63.9%
**Stage IIa**	79.0%	71.8%	63.3%	53.9%	44.2%
**Stage IIb**	67.7%	58.6%	49.0%	39.4%	30.5%
**Stage III**	48.4%	38.8%	30.0%	22.5%	16.5%

#### AHI reduction ratio

Of the baseline variables described above, stepwise multiple linear regression analysis indicated that TS Gr, AHI, ArI, Min SaO_2_ and snoring were independent predictive variables related to the AHI reduction ratio. The best predictive equation according to stepwise multiple linear regression analysis was:

$$\begin{array}{l} \text{AHI}\;\text{reduction}\;\text{ratio}=-39.464+(32.752\times \text{TS}\;\text{Gr})+(2.623\times \text{AHI})-(2.542\times \text{ArI})\\ +[1.245\times \text{Min}\;\text{Sa}{\text{O}}_{2}(\%)]-[0.599\times \text{Snoring}\;(\%)] \end{array}$$

TS Gr = 1 if TS Gr 3 or 4, TS Gr = 0 if TS Gr 1 or 2

**Fig 3:**
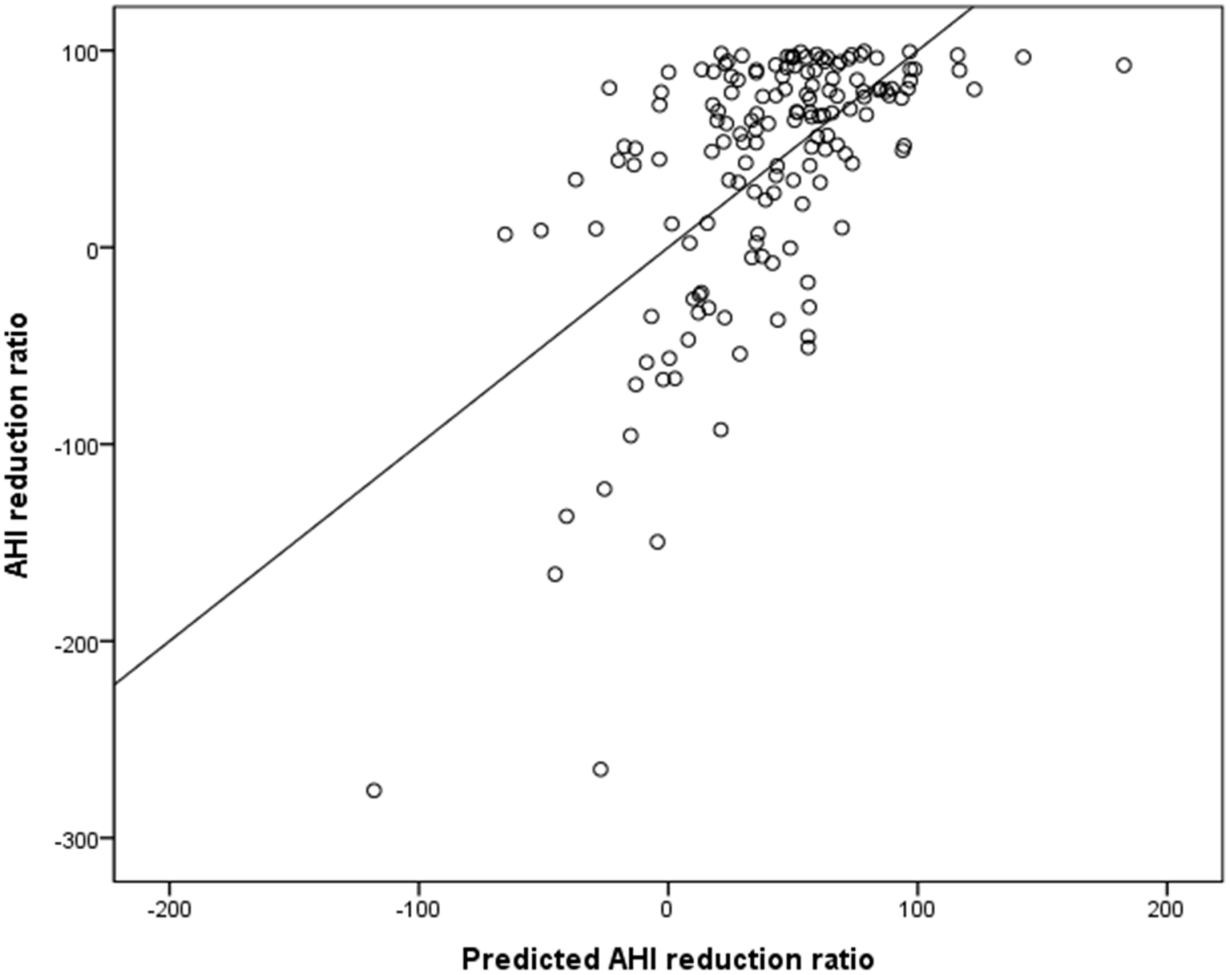
Correlation between AHI reduction ratio and predicted AHI reduction ratio (r = 0.603, P < 0.001).

Thirty-four percent of the variance in the AHI reduction ratio was explained by this equation (adjusted R^2^ = 0.342, P < 0.001) and the correlation between AHI reduction ratio and predicted AHI reduction ratio is shown in Fig 3 (r = 0.603, P < 0.001).

## Discussion

The purpose of this study was to evaluate the objective outcomes of oropharyngeal OSA surgery according to anatomical factors, and ultimately to generate predictive equation models for objective oropharyngeal OSA surgery outcomes in adults. To the best of our knowledge, this is one of the largest single institution studies performed to date, comprising 156 adult patients with pre and postoperative polysomnographic data. The results of this study can be summarized into three main categories. First, the overall success rate and AHI reduction ratio of oropharyngeal OSA surgery in our study was 55.8% (87/156) and 53.5% (20.5/38.3), respectively. Second, the success rate and AHI reduction ratio of oropharyngeal OSA surgery revealed a tendency of stratification according to anatomical structures. Thirdly, predictive equation models were developed for predicting the success rate and AHI reduction ratio before oropharyngeal OSA surgery in adult patients.

There have been numerous studies on the rates of success of oropharyngeal OSA surgery, especially UPPP.[9–12, 18–20] The rate of success for UPPP ranges from 35%–70% in non-selected patients with OSA[18, 19], and thus the success rate of 55.8% in our study fell within this range. However, when patients with OSA were appropriately selected based on anatomical structures, the success rate of UPPP increased to approximately 80%. One of the most well-known studies regarding this topic is a report by Friedman et al,[10] who indicated that stage I OSA patients have a higher success rate than stage II or III patients (80.6% vs 37.9% or 8.1%).[10] Interestingly, Li et al. compared surgical outcomes based on two staging systems (anatomy-based vs severity-based) and reported that an anatomy-based staging system is more useful in predicting the success rate for UPPP than a severity-based staging system.[12] Recently, Browaldh et al. performed a prospective randomized controlled study to evaluate the effect of UPPP in selected moderate to severe OSA patients such as Friedman stage I or II. They found that the mean AHI reduced significantly by 60% from 52.6 to 21.1 in surgical treatment group (n = 32, success rate = 59%) whereas the mean AHI reduced by 11% from 52.6 to 46.8 in control group (n = 33, success rate = 6%).[20] These results were consistent with the results of the present study, which utilized objective outcomes of oropharyngeal OSA surgery stratified or differentiated into categories according to an anatomy-based staging system.

Surgery success rate has traditionally been used as an important objective index for evaluating postoperative improvement in patients with OSA.[17] On the other hand, the AHI reduction ratio has recently been recognized as an objective index for surgical outcomes.[8] Thus, little is known about AHI reduction ratio in predicting objective surgical outcomes after OSA surgery. In the present study, the AHI reduction ratios according to stages I, II, and III were 74.1%, 49.4%, and 30.4%, respectively. Thus, we identified the possibility that the AHI reduction ratio as well as success rate could be predictors of objective outcomes after oropharyngeal OSA surgery.

It was previously established that stage II patients have large tonsils (TS G 3 or 4) and a high-level tongue (PTP G 3 or 4) or small tonsils (TS G 1 or 2) and a low-level tongue (PTP G 1 or 2).[10, 11] In the current study, we divided stage II patients into two sub-stages consisting of stage IIa (TS G 3 or 4 and PTP G 3 or 4) and IIb (TS G 1 or 2 and PTP G 1 or 2) and compared objective outcomes such as success rate and AHI reduction ratio between these two sub-stages. There was no significant difference in the success rate between stages IIa and IIb, whereas a significant difference was noted in the AHI reduction ratio between the two sub-stages. Indeed, it is thought that a modified anatomy-based staging system including two sub-stages may be helpful in evaluating objective outcomes after surgery. However, additional clinical trials are warranted to confirm that the two sub-stages may be associated with the prediction of success or AHI reduction postoperatively.

In the present study, we developed two predictive equation models for objective outcomes after oropharyngeal OSA surgery based on demographic (age, sex, BMI), anatomical (tonsil size, palate-tongue position), and polysomnographic parameters (AHI, ArI, minimum SaO_2_, snoring). Various attempts have been made to determine predictors of success in oropharyngeal OSA surgery.[18, 19, 21, 22] However, there may be some differences with respect to predictors related to improvement after oropharyngeal OSA surgery. Gislason et al. prospectively investigated favorable indications including severity of disease, degree of body weight, and radiologic findings for the success of UPPP in 34 consecutive patients with OSA and found that lower AHI and BMI are significant predictive factors.[21] Doghramji et al. tested whether preoperative Müller maneuver using fiberoptic nasopharyngoscopy and cephalometry could predict the results of UPPP, and concluded that neither approach is useful for determining successful treatment outcomes in patients.[22] Likewise, Millman et al. examined simple predictors of alleviation after UPPP and reported that a short mandibular plane-hyoid distance (MP-H ≤ 20 mm) is the single most important predictive factor of postoperative response among baseline data including anthropometry, cephalometry, and polysomnography.[19]

The present study had several limitations. First, this was a retrospective clinical analysis. Thus, we could not apply or validate the predictive equation models with another OSA patient group. Second, our results may not be universally applicable to all patients with OSA, because the parameters of our study population may be somewhat different from typical OSA populations with respect to age, sex, race, and BMI. Third, the number of women included in this study was relatively small. Finally, radiologic findings such as cephalometry were not considered in our study.

## Conclusion

The results of the present study confirmed the feasibility of objectively predicting outcomes including success rate and AHI reduction before oropharyngeal OSA surgery. The newly developed predictive equation models for objective oropharyngeal surgery outcomes may be useful for 1) planning surgical treatment and 2) improving success rates and the AHI reduction ratio via appropriate selection of adult patients with OSA. Further prospective studies will be needed to confirm and validate the usefulness of our predictive models.
